# Palliative Combined Percutaneous Balloon Aortic Valvuloplasty and Unprotected Left Main Stenting in End Stage Renal Disease

**DOI:** 10.2174/157340310790231590

**Published:** 2010-02

**Authors:** Todd A Dorfman, Raed Aqel

**Affiliations:** From the Division of Cardiovascular Disease at the University of Alabama at Birmingham, and the Division of Cardiovascular Disease, Birmingham Veterans Affair Medical Center, Birmingham, Alabama

**Keywords:** Aortic stenosis, critical left main disease, percutaneous balloon aortic valvuoplasty, unprotected left main stenting, palliative high risk percutaneous interventions.

## Abstract

With the aging population and high prevalence of atherosclerosis, an increasing number of patients presenting with heart failure and angina are found to have severe coronary artery disease and severe valvular disease. These patients tend to have multiple co-morbidities such as end stage renal disease and are considered high-risk for surgery. In patients with severe coronary artery disease, severe aortic stenosis, and heart failure with depressed left ventricular systolic function, the options are limited as they are not usually offered surgery, but palliative percutaneous high-risk procedures might be a viable alternative.

Though long term results after balloon aortic valvuolpasty are not promising, there is a role for these procedures in high-risk inoperable patients for either palliation or as a bridge to surgery. Unprotected left main percutaneous interventions are also feasible with low complication rates. This review provides mounting evidence that it is reasonable to perform combined palliative balloon aortic valvuolpasty and high-risk coronary artery stenting in certain inoperable patients. An illustrative case is presented that extends the findings of the current literature and demonstrates that combined balloon aortic valvuolpasty and left main stenting could be a safe and effective alternative in the setting of heart failure, left ventricular dysfunction, and end stage renal disease.

## CASE REPORT

A 56 year-old African American female with end stage renal disease on hemodialysis was admitted for worsening dsypnea and exertional chest pain. Her shortness of breath had begun 1-2 years ago but had progressed rapidly over the past few months. Presently she has dyspnea on exertion with minimal activity, severe orthopnea, paroxysmal nocturnal dyspnea, and marked peripheral edema and was considered to be in New York Heart Association class IV heart failure.

Past medical history was notable for congestive heart failure with preserved left ventricular (LV) function, end stage renal disease on hemodialysis, systemic arterial hypertension, and diabetes type 2. On admission the patient was on a beta blocker, ace inhibitor, non-dihydropyridine calcium channel blocker, hydralazine, a statin, and aspirin.

She was afebrile with a heart rate of 94 beats/min, blood pressure 148/75 mm Hg, and a respiratory rate 28 breaths/min with 94% oxygen saturation on room air. Her exam was otherwise notable for jugular venous pressure at about 13 cm H_2_0, delayed carotid upstrokes, and bibasilar crackles. Cardiac exam was notable for a normal S1 and a soft P2, a focal, non-displaced point of maximal impulse with no lifts, heaves, or thrills, and a III/VI systolic ejection murmur that peaked late in systole and was audible at the right upper sternal border; No rubs or gallops were noted. Extremity exam revealed marked peripheral edema.

**Fig. (1):**
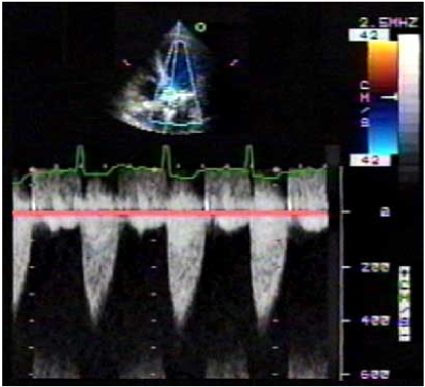
2-dimensional echocardiography reveals a calcified aortic valve with a max velocity of 4.4 m/sec, a mean velocity of 3.4 m/sec, a max gradient of 73 mm Hg with a mean gradient of 47 mm Hg, and an aortic valve area of 0.7 cm2.

Transthoracic echocardiography revealed a LV ejection fraction of 35%, preserved right ventricular function, mild mitral regurgitation, moderate tricuspid regurgitation, a heavily calcified aortic valve with three leaflets, and severe aortic stenosis (AS) (Fig. **1**). Her pulmonary artery systolic pressure was severely elevated at 86 mm Hg and was calculated by the peak tricuspid regurgitation jet using the simplified Bernoulli equation.

**Fig. (2):**
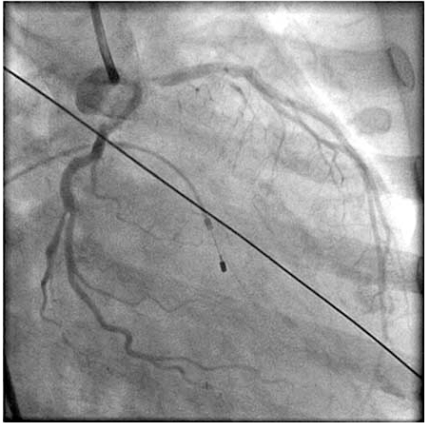
Coronary angiography in the RAO caudal projection demonstrates a distal 60-70% LM stenosis, and a 95% ostial stenosis of the LAD.

Left and right heart catheterizations were performed and were remarkable for severe ostial left anterior descending (LAD) coronary artery disease with distal left main (LM) involvement (Fig. **2**), minimal left circumflex and right coronary artery disease, a right atrial pressure of 14 mm Hg, pulmonary artery pressure of 67/29 with a mean of 45 mm Hg, and a pulmonary capillary wedge pressure of 23 mm Hg; Left ventricular pressure was 143/18 mm Hg, and aortic pressure was 85/43 mm Hg with an aortic valve mean gradient of 56 mm Hg.

Given the constellation of severe heart failure, severe AS, and severe ostial LAD disease with distal LM involvement, cardiovascular surgery was consulted for a potential aortic valve replacement (AVR) and coronary artery bypass grafting, but the patient was considered a poor surgical candidate due to end stage kidney disease and elevated pulmonary arterial pressures and was treated initially with optimal medical management. 

**Fig. (3):**
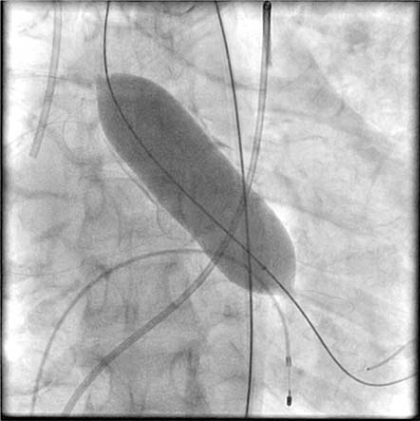
Using rapid right ventricular pacing at 160 beats/minute, BAV was performed with a 25 mm x 6.0 cm balloon dilation catheter (Z-Med IITM). After 2 inflations across the aortic valve, the waist on the balloon disappeared, and the LV-aortic gradient decreased from 56 to 24 mm of Hg.

**Fig. (4):**
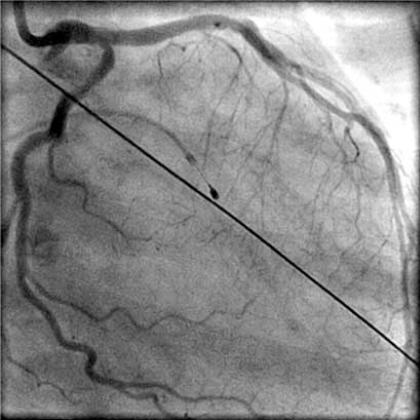
Following LM and LAD angioplasty and stenting, the kissing balloon technique was performed on the LAD and left circumflex artery with excellent results.

However, the patient went into cardiogenic shock and was unable to tolerate dialysis. Emergent measures were discussed, and after much deliberation between the patient and her family, the patient elected to undergo high-risk percutaneous balloon aortic valvuloplasty (BAV) and stenting of an unprotected LM and LAD. BAV was performed with a 25 mm x 6.0 cm balloon dilation catheter (Z-Med II^TM^), which was placed across the aortic valve. After 2 inflations, there was a marked reduction in the LV-aortic gradient (Fig. **3**). After the second inflation, the patient went into ventricular fibrillation and was stabilized with multiple DC cardioversions and emergent intubation with mechanical ventilation. Coronary angioplasty and stenting of the unprotected LM and LAD artery were performed (Fig. **4**).

The patient was extubated within 48 hours, and her volume status was optimized prior to being discharged to home in stable condition. At 6-month follow-up, she was in New York Heart Association class II heart failure with a markedly improved exercise capacity and was free of chest pain.

## DISCUSSION

The incidence of co-existing severe AS and LM disease is unknown, but is believed to be rising and presents a tremendous challenge to patients and clinicians [1]. If the patient is a poor surgical candidate due to multiple comorbidities, the remaining options are primarily palliative in nature.

LM stenting is a viable option in inoperable candidates and is associated with high rates of technical success, low procedural risk, and low rates of cardiac death (11.9%) at 3 year follow-up [2]. The need for repeat revascularization might be as high as 23.9% according to a recent experience including 67 patients [2]. 

Recently a large non-randomized trial compared 1102 patients with unprotected LM disease who underwent stenting and 1138 patients who underwent coronary artery bypass grafting. At 3 years, the risk of restenosis requiring target-vessel revascularization was significantly higher in patients that received stents than in those who underwent surgical revascularization, but there was no significant difference in the risk of death or in the cumulative endpoint of death, myocardial infarction, or stroke between the two groups. While the need for repeat revascularization was greater after bare metal stent implantation as compared to drug-eluting stents, there was a slight increase in death and in the composite end point in those patients that received drug-eluting stents [3]. Additional studies have also compared drug-eluting stents and bare metals stents in the setting of unprotected LM interventions. 220 patients with unprotected LM disease underwent drug-eluting stent implantation and were compared with a historical control, which included 224 patients who received bare metal stents. At 15-month follow-up, the incidence of target-lesion revascularization, major adverse cardiac events, and cardiac death were significantly lower after drug-eluting stent implantation [4]. Drug-eluting stents and bare metal stents have even been compared in patients with severe unprotected LM coronary artery disease in the setting of an acute coronary syndrome, and while drug-eluting stent implantation resulted in less myocardial infarction and less target-vessel revascularization than bare metal stents, mortality was similar between the two groups [5]. A randomized trial which included over 600 patients who underwent unprotected LM stenting showed that there was no clinical or angiographic difference observed after implantation with either a Paclitaxel or a Sirolimus stent [6].

Indications for percutaneous BAV are limited due to short-term benefit, high complication rates, and a high risk of restenosis [7], but it remains a reasonable option in certain patients. BAV fractures the calcific deposits on the valve leaflets, minimally stretches the annulus, and mildly separates the commissures [8, 9]. This results in a moderate increase in aortic valve area, a modest reduction in the transvalvular pressure gradient, and a mild increase in LV systolic function [8, 9]. Following BAV the aortic valve area is infrequently greater than 1.0 cm^2^, and BAV is associated with a 10% rate of severe acute complications. While the majority of patients report symptomatic improvement after BAV, there is a tendency for clinical deterioration within 6-12 months due to a high likelihood of restenosis [8].

144 patients with severe AS were treated with BAV after they were not offered AVR due to high surgical risk. 57% did not require additional treatment (group 1). Repeat BAV was performed in 19% of patients secondary to restenosis (group 2), and AVR was done in the remaining 24% of cases (group 3). Survival at 3 years from the date of the last intervention was 13%, 20%, and 75% for group 1, 2, and 3 respectively [7].

Certainly, AVR is superior to BAV and is indicated for symptomatic patients with severe AS and in patients with severe AS and decreased LV systolic function (EF <50%). According to the present ACC/AHA guidelines, BAV is not recommended as an alternative to AVR for patients with severe AS, but is a reasonable option as a bridge to AVR in hemodynamically unstable patients who are at high risk for surgery (Class IIb) [8]. BAV is frequently used as a bridge to AVR in patients with severe AS and New York Heart Association Class III-IV heart failure as improved hemodynamics likely decrease the risk of surgery [7, 8].

BAV is also a class IIb indication by the present ACC/AHA guidelines for palliation when surgery cannot be performed secondary to serious co-morbidities [8], but the indications for performing palliative procedures is unclear. Although there is minimal data that these high-risk procedures prolong life [8], these inoperable patients have limited options, and there is no medical treatment available that improves survival [8]. In fact, medications that alleviate pulmonary edema by decreasing preload and central volume can also result in excessive reductions in preload and an unsafe drop in cardiac output [8].

Percutaneous AVR is a developing technology that might be an alternative treatment in high-risk inoperable patients with severe AS, but its safety and efficacy remain controversial. A self-expanding prosthetic valve can be deployed percutaneously via a retrograde approach in approximately 84-93% of patients. When successfully implanted, it increases aortic valve area, reduces transvalvular gradients, and improves New York Heart Association functional class, but major adverse cardiac events occurred in as many as 32% of patients at short term follow-up, which included death in 20% [10-12]. The largest trial to date which included 86 patients reported a procedural mortality of 6%, a 30-day mortality of 12%, and a cumulative incidence of death, stroke, and myocardial infarction of 22% [12]. Percutaneous AVR is also limited by bleeding (24%) and paravalular leakage (48%) and might be less successful in the setting of a bicuspid aortic valve [13]. While previous trials investigating percutaneous AVR are small, non-randomized, and lack long term follow-up, there still is enthusiasm for this emerging technology; however, its role in clinical practice remains limited.

Patients with symptomatic co-existing severe coronary artery disease, severe AS, and heart failure are frequently deemed to be poor surgical candidates, but these high-risk patients have percutaneous options. According to several case reports, BAV can be safely performed 1-2 weeks prior to percutaneous coronary interventions (PCI) in patients with severe AS, refractory heart failure with LV dysfunction, and coronary artery disease who are not considered to be surgical candidates for AVR and coronary artery bypass grafting [1, 14, 15]. For example, an elderly male with severe AS, chest pain, and NYHA class IV heart failure underwent BAV followed by successful LM stenting within 1 week [1].

There is even some data supporting the safety of performing these high-risk interventions at the same time. In the Spanish literature, a morbidly obese 65-year-old female successfully underwent combined BAV and ostial left circumflex stenting prior to bariatric surgery. AVR and coronary artery bypass grafting were then performed 4 months later [16].

In addition to case reports, several small cohorts have also demonstrated the safety and utility of combining palliative percutaneous BAV with coronary artery interventions [1, 14, 15, 17, 18]. Combined BAV with single vessel coronary angioplasty was performed in 9 patients with critical AS, chest pain, and heart failure. 8 out of 9 patients had marked improvement in symptoms at discharge. PCI was done on the LAD (n = 3), left circumflex (n = 3), right coronary artery (n = 2), and a saphenous vein graft to the right coronary artery (n = 1). Complications included 2 groin hematomas, 1 transient left bundle branch block, and 1 case of transient atrial fibrillation [17]. Combined BAV and PCI were performed successfully in 17 other patients at a different medical center. PCI was done prior to BAV in 13 patients and after BAV in another 4 cases without periprocedural mortality, myocardial infarction, or stroke [18]. While these single center experiences demonstrated that performing combined BAV and PCI in inoperable patients is a viable option, these studies failed to include LM interventions, and to our knowledge, our case is the first to demonstrate the safety of combined BAV and LM stenting in the English literature. Further studies are clearly warranted.

Furthermore, the decision to treat the valve first or the coronaries first has not been addressed and remains unclear. In our experience we believe that clinical presentation and anatomy should dictate management. For example, patients presenting primarily with heart failure and volume overload in the setting of severe untreated AS appear to have a dismal prognosis when they suffer myocardial ischemia during complex PCI. Thus, we recommend performing percutaneous BAV prior to PCI in these patients as this approach likely reduces the risk of the procedure by unloading the LV, decreasing diastolic wall stress, prolonging diastolic perfusion time, and increasing coronary flow reserve. This approach also allows more time for complicated PCI especially in the setting of complex unprotected LM stenting. However, if an inoperable patient with severe AS, congestive heart failure, and severe CAD presents with an acute coronary syndrome and has anatomy suitable for uncomplicated stenting, perhaps PCI should be performed prior to BAV. Nevertheless, the question as to which should be addressed first remains unanswered.

## CONCLUSIONS

Combined palliative BAV and LM stenting is a viable option in inoperable patients with severe AS, severe coronary artery disease, and heart failure with depressed LV systolic function. While AVR and coronary artery bypass grafting remain the superior option, it is reasonable to offer these high-risk patients a combined palliative percutaneous procedure for symptomatic relief. Given the paucity of data, certainly further investigation is warranted as to whether the aortic valve or coronary artery should be addressed first.

## ABBREVIATIONS

LV: = Left ventricular
AS: = Aortic stenosis
LAD: = Left anterior descending artery coronary artery
LM: = Left main
AVR: = Aortic valve replacement
BAV: = Balloon aortic valvulplasty
PCI: = Percutaneous coronary interventions
